# An emboligenic pulmonary abscess leading to ischemic stroke and secondary brain abscess

**DOI:** 10.1186/1471-2377-12-133

**Published:** 2012-11-05

**Authors:** Philipp Albrecht, Mark Stettner, Leila Husseini, Stephan Macht, Sebastian Jander, Colin Mackenzie, Ulrike Oesterlee, Philipp Slotty, Axel Methner, Hans-Peter Hartung, Orhan Aktas

**Affiliations:** 1 Department of Neurology, Medical Faculty, Heinrich-Heine University Düsseldorf, Düsseldorf, Germany; 2 Department of Diagnostic and Interventional Radiology, Medical Faculty, Heinrich-Heine University Düsseldorf, Düsseldorf, Germany; 3 Department of Medical Microbiology and Hospital Hygiene, Medical Faculty, Heinrich-Heine University Düsseldorf, Düsseldorf, Germany; 4 Department of Cardiology, Medical Faculty, Heinrich-Heine University Düsseldorf, Düsseldorf, Germany; 5 Department of Neurosurgery, Medical Faculty, Heinrich-Heine University Düsseldorf, Düsseldorf, Germany

## Abstract

**Background:**

Ischemic stroke by septic embolism occurs primarily in the context of infective endocarditis or in patients with a right-to-left shunt and formation of a secondary cerebral abscess is a rare event. Erosion of pulmonary veins by a pulmonary abscess can lead to transcardiac septic embolism but to our knowledge no case of septic embolic ischemic stroke from a pulmonary abscess with secondary transformation into a brain abscess has been reported to date.

**Case presentation:**

We report the case of a patient with a pulmonary abscess causing a septic embolic cerebral infarction which then transformed into a cerebral abscess. After antibiotic therapy and drainage of the abscess the patient could be rehabilitated and presented an impressive improvement of symptoms.

**Conclusion:**

Septic embolism should be considered as cause of ischemic stroke in patients with pulmonary abscess and can be followed by formation of a secondary cerebral abscess. Early antibiotic treatment and repeated cranial CT-scans for detection of a secondary abscess should be performed.

## Background

Cerebral abscess is a rare but severe disease and can result in severe disability or even death, especially if misdiagnosed or improperly managed. In most cases a brain abscess is preceded by a phase of cerebritis with headache followed by symptoms of increased intracranial pressure (nausea, vomiting, somnolence) and only about 30% of patients present initial focal neurological deficits [1]. Most often brain abscesses arise from direct penetration after brain injury, operation, sinusitis or otitis. About 20-30% are caused by metastatic spread which most frequently originates from infectious endocarditis (2.8% of these patients [2,3]) or from paradoxical embolism in patients with a right-to-left shunt [1]. The rare metastatic spread from a pulmonary abscess has however been described in the literature [4]. Embolisation of septic material e.g. in infective endocarditis is a known reason for strokes but only in very rare cases is the condition complicated by formation of a secondary brain abscess [5,6].

In emergency situations, one should bear in mind that systemic thrombolysis of septic-embolic strokes e.g. in infective endocarditis has been reported to be effective but associated with an increased risk of haemorrhage [7-9].

## Case presentation

**Figure 1:**
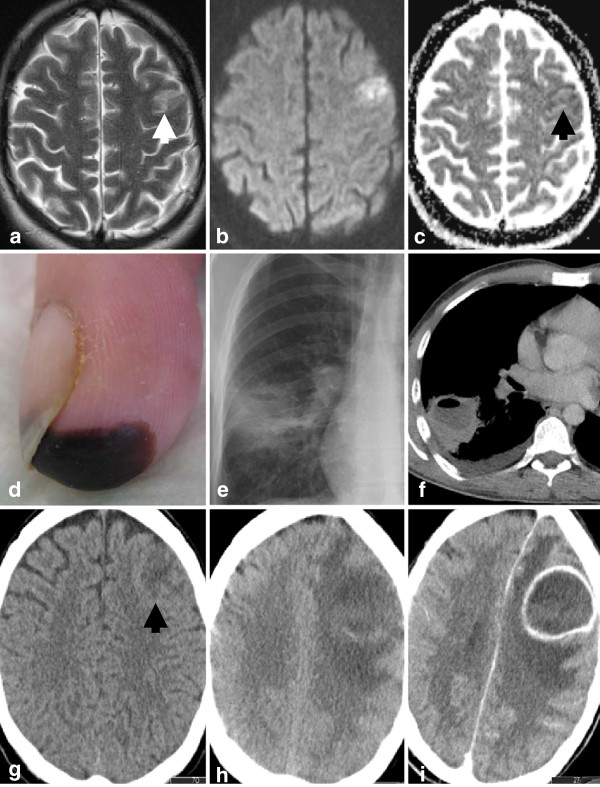
**a) T2 weighted axial image displayed a hyperintense cortical signal alteration in two adjacent gyri of the left supplementary motor cortex (white arrow).b**) This lesion was hyperintense in the axial diffusion weighted image (b = 1000 sec/mm^2^. Although it showed only minimally lowered ADC values (Apparent Diffusion Coefficient, black arrow, **c**), these findings were primarily consistent with an acute infarction. **d**) The index finger of his left hand showed a typical Janeway lesion highly indicative of a septic-embolic focus. Chest X-Ray (**e**) and computed tomography (**f**) showed a large fluid and air containing process in the right lower lobe consistent with a septic lung abscess. **g**) A computed tomography two days later revealed a now well demarcated lesion (native scan in axial orientation, black arrow). **h**) A further nine days later, after clinical deterioration a repeated scan revealed a large left frontal mass (native scan) with ring-like enhancement after i.v. administration of iodine contrast media (**i**). The findings were now typical for a brain abscess.

We report the case of a 35-year-old man with a 21-year history of intravenous heroin abuse who developed moderate global aphasia on the day of admission. Cerebral magnetic resonance imaging (MRI) was in line with an acute ischemic infarction in the left supplementary motor cortex (Figure 1a-c). 4.5 h after symptom onset, systemic thrombolysis was no longer a therapeutic option. Laboratory parameters on admission were consistent with an acute bacterial infection with a C-reactive protein level of 23.0 mg/dl and leukocytosis of 21.3x10^3^/μl. Detailed clinical examination revealed a Janeway lesion on the fingertip of his left hand (Figure 1d), which was highly indicative of a septic-embolic focus as the cause also of the ischemic stroke. Transthoracic as well as transesophageal echocardiography provided no evidence for an endocarditis or cardiac right-to-left shunt and repeated aerobic and anaerobic blood cultures came out negative. Magnetic resonance angiography and doppler/duplex-sonography revealed no relevant stenoses of the cervical or cerebral arteries. Chest X-ray, however, showed a large fluid and air containing lesion in the right lower lobe (Figure 1e) which was confirmed as a lung abscess by computed tomography (CT, Figure 1f). There were no further lesions suspicious of septic emboli in the high-resolution CT-scan or other chest X-rays. Antibiotic treatment with piperacillin/tazobactam 3x4.5g/d was immediately initiated. Bronchoscopy confirmed a pulmonary abscess; culture of the aspired purulent liquid revealed no bacterial growth. Tuberculosis was considered unlikely due to the negative interferon-gamma release assay, negative Ziehl Neelsen stain and cultures of sputum, urine, and gastric juice. HIV serology was also negative. As a right-to-left shunt was excluded and echography gave no evidence of a tricuspid endocarditis, we considered the pulmonary abscess to be a consequence of the intravenous heroin abuse.

As contrast transcranial Doppler sonography [10] and echocardiography showed no right-to-left shunt an erosion of a pulmonary vein by the pulmonary abscess and consecutive transcardiac embolisation was thought to be the most probable cause of the ischemic stroke and the Janeway lesion. 72 h monitoring of blood pressure and ECG and a formal 24 h holter ECG gave no evidence of arterial hypertension or relevant cardiac arrhythmia; serum cholesterol, LDL and HDL were normal.

A control CT scan two days after admission revealed a round, well demarcated lesion at the location of the initial stroke (Figure 1g). 11 days after admission the neurological status deteriorated and the patient developed a complete paralysis of his right arm and global aphasia. On the same day a CT scan revealed a large left frontal mass with ring-enhancement of contrast media typical for a cerebral abscess (Figure 1h-i). The patient was immediately transferred to the neurosurgical department where the abscess was drained and rinsed. The antibiotic treatment was changed to metronidazol 3x500mg/d, vancomycin 2x1g/d, and ceftriaxone 2g/d. Cultures of the abscess content revealed *Fusobacterium nucleatum* which was sensitive to ampicillin/sulbactam and metronidazol. The drainage was removed after one week but as the size of the abscess cavity and oedema increased in a control CT scan a new drainage was placed another week later and the abscess rinsed daily. CT scan showed no further progression thus the drainage could be removed and the patient was transferred to a rehabilitation centre where antibiotic treatment with ampicillin/sulbactam 3x3g/d and metronidazole 2x500mg/d was continued for another six weeks. During rehabilitation the neurological status improved, the patient was able to walk and speak again and could be discharged after seven weeks with only a latent paresis of his right arm and a slight incomplete aphasia which were both barely noticeable on examination corresponding to a modified Rankin scale of 1.

## Conclusion and discussion

This case illustrates that septic embolism should be considered as a cause of ischemic stroke in patients with pulmonary abscess. Even though paradox embolism due to cardiac or pulmonary right-to-left-shunting is a common reason of stroke in patients with i.v. drug abuse we do not believe this was the case in our patient as one would expect a shunt to persist and be detectable by the transoesophagic echocardiography or the contrast transcranial Doppler shunt-detection exam which togeather have a very high sensitivity (reviewed in [11]). We therefore believe that an erosion of a pulmonary vein by the pulmonary abscess and consecutive transcardiac embolisation of septic material was the most probable cause of the ischemic stroke and the Janeway lesion of our patient.

Our case suggests that such patients should be put on broad-spectrum antibiotic therapy and be closely monitored with repeated cerebral imaging to identify the possible formation of a secondary abscess after stroke. An earlier imaging by MRI or CT with contrast medium may have showed the abscess even before clinical deterioration which could have led to earlier escalation of the antibiotic therapy and allowed for earlier neurosurgical treatment ameliorating the prognosis.

Furthermore this case highlights the need for a very rapid microbiological examination of good quality specimens to obtain an antibiogram and switch to a focused antibiotic therapy. We suspect that the *fusobacterium* in our case did not survive the transport in the specimen from the lung.

## Consent

Written informed consent was obtained from the patient for publication of this Case report and any accompanying images. A copy of the written consent is available for review by the series editor of this journal.

## Competing interests

The authors declare that they have no competing interests or financial disclosures.

## Authors’ contributions

PA has analyzed and interpreted the case, drafted the manuscript and submitted the final revised version. All authors have made substantial contributions to interpretation of the case, they have revised the manuscript critically for important intellectual content and have given their final approval of the version to be published.

## Pre-publication history

The pre-publication history for this paper can be accessed here:

http://www.biomedcentral.com/1471-2377/12/133/prepub
